# Rate of osmotic pressure change in drying saliva microdroplets drives inactivation of model respiratory bacteria

**DOI:** 10.1128/msphere.00594-26

**Published:** 2026-10-07

**Authors:** Taylor Medina, Beiping Luo, Thomas Peter, Htet Kyi Wynn, Tamar Kohn

**Affiliations:** 1 Laboratory of Environmental Virology, School of Architecture, Civil and Environmental Engineering, École Polytechnique Fédérale de Lausanne 111839 , Lausanne, Switzerland; 2 Institute for Atmospheric and Climate Science, ETH Zürich 536522, Zürich, Switzerland; The University of Arizona, Tucson, Arizona, USA

**Keywords:** osmotic pressure, IRP, respiratory droplet, *E. coli*, *S. epidermidis*

## Abstract

Transmission of respiratory pathogens depends on their ability to remain viable in drying respiratory droplets, yet the physicochemical drivers of bacterial inactivation during droplet evaporation remain poorly quantified. This study combines controlled droplet experiments with physicochemical modeling to investigate how osmotic pressure dynamics influence bacterial survival. Using *Escherichia coli* and *Staphylococcus epidermidis* as gram-negative and gram-positive surrogates, respectively, we measured viability loss in artificial saliva droplets dried at multiple relative humidities, and reconstructed the time-resolved osmotic pressure using the respiratory aerosol model. Both organisms remained stable while droplets were liquid and osmotic pressure was low but lost viability as decreasing water activity during drying produced rapid increases in osmotic pressure. For droplets that effloresced, these changes were most pronounced around efflorescence. The extent of inactivation scaled log-linearly with the rate of osmotic pressure change: *E. coli* decayed faster than *S. epidermidis*, and relationships derived in artificial saliva predicted survival in independent phosphate-buffered saline experiments. A lower relative humidity produced a more rapid increase in osmotic pressure and greater inactivation. These results identify the rate of osmotic pressure change during respiratory droplet drying as a quantitative predictor of bacterial survival across different solution matrices and drying conditions.

**IMPORTANCE:**

The risk of infection by respiratory pathogens depends on how long microorganisms remain viable in respiratory particles after exhalation, yet the physical mechanisms controlling bacterial survival during droplet drying are not well defined. Evaporation of respiratory droplets concentrates salts and can impose sudden and extreme osmotic stress on microbes, but this process has been difficult to quantify because osmotic pressure cannot be measured directly inside microscopic droplets. Integration of droplet experiments with a physicochemical aerosol model shows that bacterial inactivation is governed primarily by the rate of osmotic pressure increase during droplet drying rather than by static values of humidity or solute concentration alone. This mechanism explains why rapid drying produces strong inactivation.

## INTRODUCTION

Respiratory pathogens, including viruses, bacteria, and fungi, are expelled from infected individuals in infectious respiratory particles (IRPs), which span a continuum of particle sizes (1). Following release, these particles undergo rapid evaporation and associated physicochemical changes that influence microbial viability. The ability of microorganisms to remain viable during the drying of respiratory fluids is therefore an important factor influencing respiratory pathogen transmission. However, the mechanisms modulating bacterial viability during droplet drying remain only partially understood.

A known driver of bacterial viability loss in IRPs is the changing salt concentration (2, 3). After IRPs are exhaled, they are subject to evaporation, which is dependent on the ambient relative humidity (RH). As evaporation proceeds and the dissolved salts become supersaturated, the droplets may undergo efflorescence, the phase transition in which dissolved salts crystallize from an evaporating aqueous droplet. Efflorescence occurs when ambient relative humidity falls below the efflorescence relative humidity (4). However, the presence of a nucleating agent, such as insoluble particles or certain biological materials, can promote heterogeneous nucleation and cause efflorescence to occur at a higher RH (5). During the drying process, salt concentrations can reach high supersaturations, but the concentration drops back to saturation levels once efflorescence takes place (5).

Although the specific response to changing salt concentrations in IRPs varies between pathogen types, bacterial inactivation can, at least in part, be attributed to hyperosmotic shock resulting from rapid changes in water activity in the surrounding solution as droplets approach equilibrium with the surrounding relative humidity (6). This shock arises from a rapid change in solute concentrations in the surrounding solution to levels that exceed those of the intracellular environment. As evaporation proceeds, the increasing solute concentration reduces the water activity (*a*_*w*_), a dimensionless quantity relative to pure water that reflects the chemical potential of water (7). This leads to a rise in the osmotic pressure (Π), a thermodynamic measure of a solution’s tendency to draw in water relative to pure water, proportional to *−*ln(*a*_*w*_) (7, 8). For a bacterium within the liquid phase of a droplet, this defines the extracellular osmotic environment (Π_droplet_). Increases in extracellular osmotic pressure drive water efflux from the cytoplasm, resulting in a reduction in turgor pressure (*P*_turgor_), the mechanical force generated by the difference between the intracellular (Π_bacteria_) and extracellular osmotic pressures (*P*_turgor_ *=* Π_bacteria_ *–* Π_droplet_) (9, 10). Turgor pressure is essential for maintaining cellular shape and serves as a signal for regulating the expression of osmoregulatory genes (10). Rapid increases in Π_droplet_ can cause a sharp decline in *P*_turgor_ before osmoadaptive mechanisms can restore intracellular osmotic balance. This transient loss of turgor leads to membrane deformation, mechanical stress on the cell envelope, and ultimately loss of viability, thereby linking evaporation dynamics to bacterial viability loss in IRPs.

*Escherichia coli* and *Staphylococcus epidermidis* represent gram-negative and gram-positive bacteria with distinct ecological niches and physiological adaptations. *E. coli* is primarily associated with aqueous environments such as the gastrointestinal tract, whereas *S. epidermidis* is adapted to the human skin, where it is routinely exposed to desiccation and osmotic stress. These differences are reflected in their osmotic stress responses. *E. coli* and other gram-negative bacteria maintain a turgor pressure of about 2–4 atm through molecular osmoadaptation mechanisms and can tolerate transient osmotic pressure increases up to 10–20 atm (11). However, viability depends not only on the magnitude of osmotic pressure increase, but also on the rate of increase, as rapid increases in Π_droplet_ produce abrupt reductions in *P*_turgor_ that the bacterium cannot compensate for *E. coli* can only fully recover from shocks up to 22 atm within 10–15 min (7, 12). While the initial response to hyperosmotic stress is initiated within 1–2 min, full osmoadaptive gene expression requires approximately 1 h, depending on physiological state and media composition (13). The long response time renders gram-negative bacteria generally intolerant to the rapid osmotic shocks seen during droplet drying.

In contrast, gram-positive bacteria generally exhibit higher resistance to osmotic and desiccation stress (14). This resilience is attributed to their thicker peptidoglycan layer, which provides additional mechanical strength and allows the cell wall to sustain higher turgor pressures, often exceeding 20 atm (15). Elevated turgor pressure, together with the rigid peptidoglycan matrix, buffers the cell against external osmotic shock by reducing the relative change in transmembrane pressure and limiting membrane deformation. This mechanical stabilization mitigates cell lysis and structural damage under high osmotic conditions. As a result, gram-positive bacteria typically show slower inactivation rates under drying conditions compared to gram-negative species, which are more vulnerable to rapid water loss and membrane tension spikes (10).

Despite the recognized importance of osmotic pressure as a driver of bacterial inactivation in IRPs (6, 7, 16–18), there is currently no quantitative approach to describe the dependence of inactivation kinetics on osmotic pressure within these particles. This is partly due to the fact that direct internal measurements of osmotic pressure are not feasible. This study addresses this fundamental gap by combining empirical data on bacterial inactivation with a physicochemical droplet model to estimate osmotic pressure during drying.

We hypothesize that bacterial viability loss during respiratory droplet drying is governed primarily by the rate of osmotic pressure increase as droplets approach equilibrium with the surrounding relative humidity. *Escherichia coli* (gram-negative) and *Staphylococcus epidermidis* (gram-positive) were selected as representative bacteria with distinct cell envelope architectures. Both organisms are used as model microorganisms because they can be handled under lower biosafety requirements while retaining many of the structural and physiological characteristics relevant to environmental persistence and stress responses of pathogenic bacteria (19, 20). This study first quantifies the inactivation kinetics of *E. coli* and *S. epidermidis* in 1 µL deposited artificial saliva droplets under controlled RH conditions and then employs the physicochemical model respiratory aerosol model (ResAM) to estimate osmotic pressure over time inside the droplet throughout droplet drying (3). This integrated approach allows the establishment of a quantitative correlation between modeled change in osmotic pressure as droplets approach equilibrium and microbial viability in drying respiratory droplets, providing mechanistic insight into bacterial survival.

## RESULTS

### Bacterial inactivation and osmotic pressure in drying saliva droplets

To determine how relative humidity (RH) influences bacterial survival during droplet drying, *E. coli* and *S. epidermidis* suspended in 1 μL artificial saliva droplets were deposited on hydrophobic-coated 96-well plates and were dried at low (30%), medium (50%), and high (70%) RH. Bacterial viability in the droplets was monitored for 60 min at 30% RH for *E. coli* and for 120 min for the rest of the experiments. Parallel simulations using ResAM modeled the evolution of Π_droplet_ based on droplet composition, RH, and observed droplet radius.

**Fig 1:**
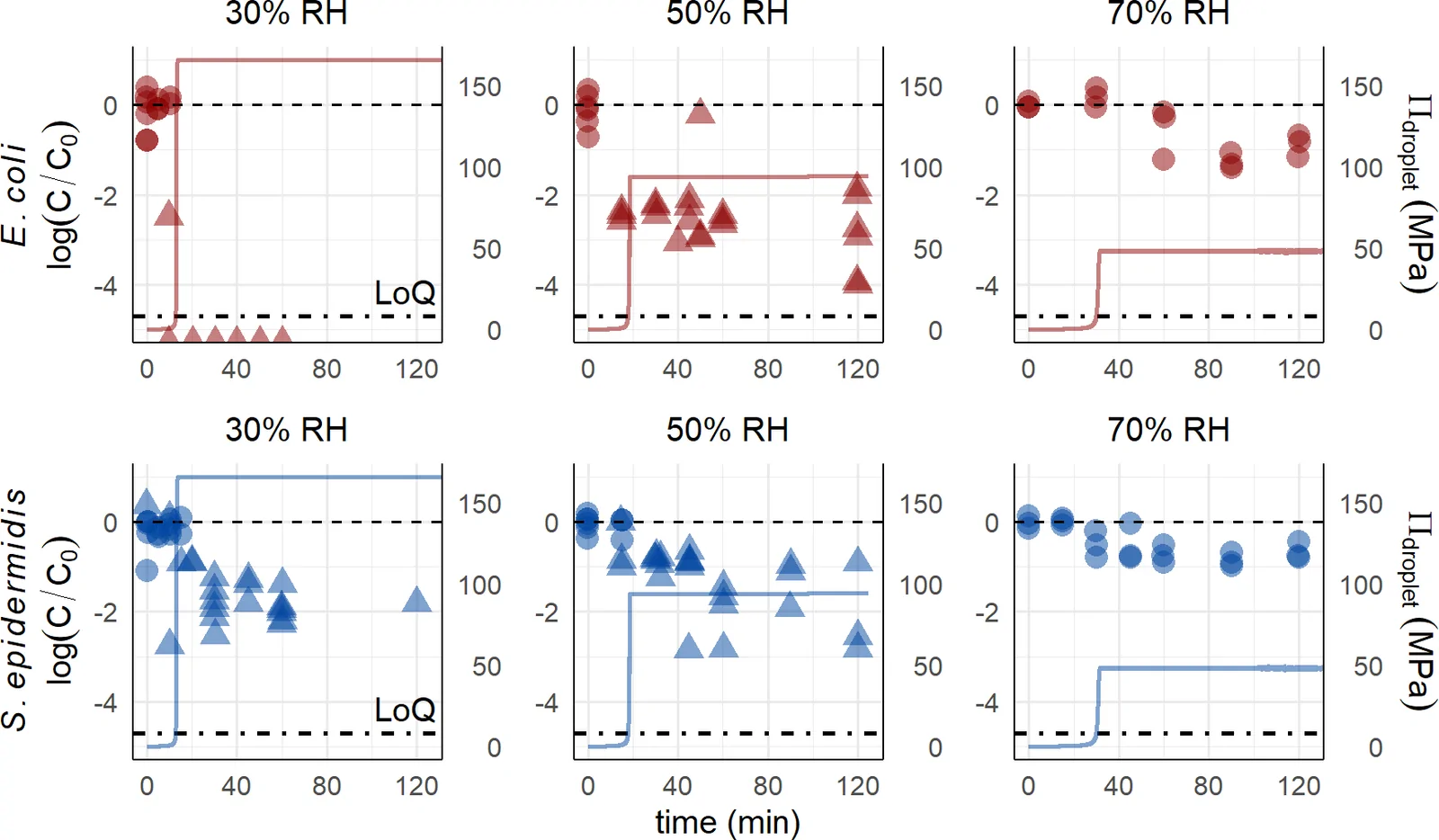
Inactivation of *E. coli* (red) and *S. epidermidis* (blue) in 1 μL artificial saliva droplets dried at 30%, 50%, and 70% relative humidity (RH) with modeled osmotic pressure (Π_droplet_). The left axis represents the log_10_ reduction of the bacteria, while the right axis shows the modeled osmotic pressure Π_droplet_ (MPa) shown as a solid line. Circles denote liquid droplets, and triangles represent effloresced droplets as determined by visual inspection. The starting titers of *E. coli* and *S. epidermidis* were both 10^8^ CFU/mL. LoQ, limit of quantification (−4.7 log_10_).

Figure 1 shows bacterial inactivation curves together with the modeled osmotic pressure. The osmotic pressures reported here are referenced to pure water and capture the magnitude and rate of increase in the extracellular osmotic environment, thereby representing the osmotic shock experienced by the bacterium.

During the initial drying phase, osmotic pressure changed only slightly as water evaporated (Fig. 1). For droplets that effloresced (30% and 50% RH), osmotic pressure increased sharply as solutes approached supersaturation, followed by a brief decrease associated with crystallization and a subsequent rise to the equilibrium osmotic pressure determined by RH. The modeled dynamics therefore exhibit a characteristic two-stage structure (not visible at the time scale in Fig. 1, but shown in a zoom-in in Fig. S1 for artificial saliva at 50% RH). For droplets that remained liquid throughout the experiment (70% RH), osmotic pressure increased in a single stage as water activity decreased until the droplets reached equilibrium with the surrounding RH. The largest change in osmotic pressure (ΔΠ) occurred at 30% RH (162.5 MPa), followed by 50% RH (94.0 MPa) and 70% RH (49.4 MPa).

Across all RH conditions, both organisms remained stable when Π_droplet_ was low and after the droplets had reached equilibrium, even when Π_droplet_ was high. Inactivation occurred only during the sharp increase in osmotic pressure resulting from the rapid concentration of solutes as droplet water activity decreased toward its equilibrium with the ambient RH. For *E. coli*, the magnitude of inactivation depended strongly on RH: it exceeded the quantification limit (>5 log_10_ reduction) at 30% RH, was approximately 3.8 log_10_ at 50% RH, and approximately 1 log_10_ at 70% RH. *S. epidermidis* followed the same qualitative pattern but experienced less inactivation, with approximately 1.7 log_10_ decay at 30% RH, 1.5 log_10_ at 50% RH, and 0.7 log_10_ at 70% RH. Analysis of covariance accounting for sampling time confirmed that *S. epidermidis* exhibited significantly greater survival than *E. coli* at both 30% RH (estimated difference = 3.2 log_10_, *P* < 0.0001) and 50% RH (estimated difference = 0.9 log_10_, *P* < 0.0001). At 70% RH no significant difference in survival was observed between species after 30 min (estimated difference = −0.07 log_10_, *P* = 0.782).

In contrast to viability, the concentration of bacterial genome copies remained relatively constant throughout the experiment (Fig. S2), indicating that the observed decay resulted from inactivation rather than adsorption of bacteria to the well plates. Neither *E. coli* nor *S. epidermidis* showed significant decay when held for 24 h in bulk saturated NaCl solutions, where no changes in osmotic pressure occur (Fig. S3).

Furthermore, to distinguish the inactivating effects of osmotic shock from those of crystal formation, droplet experiments were performed in aqueous polyethylene glycol 200 (PEG-200), which remains liquid over the experimental water activity range (21). Bacterial inactivation of 3.5 log_10_ was observed for both organisms in droplets at 50% RH (corresponding to an osmotic pressure change of 94.0 MPa), despite the absence of efflorescence (Fig. S4). This finding demonstrates that crystal formation is not necessary for inactivation and supports the hypothesis that the rapid increase in osmotic pressure is the dominant driver of bacterial viability loss.

### Association between inactivation and rate of osmotic pressure change

**Fig 2:**
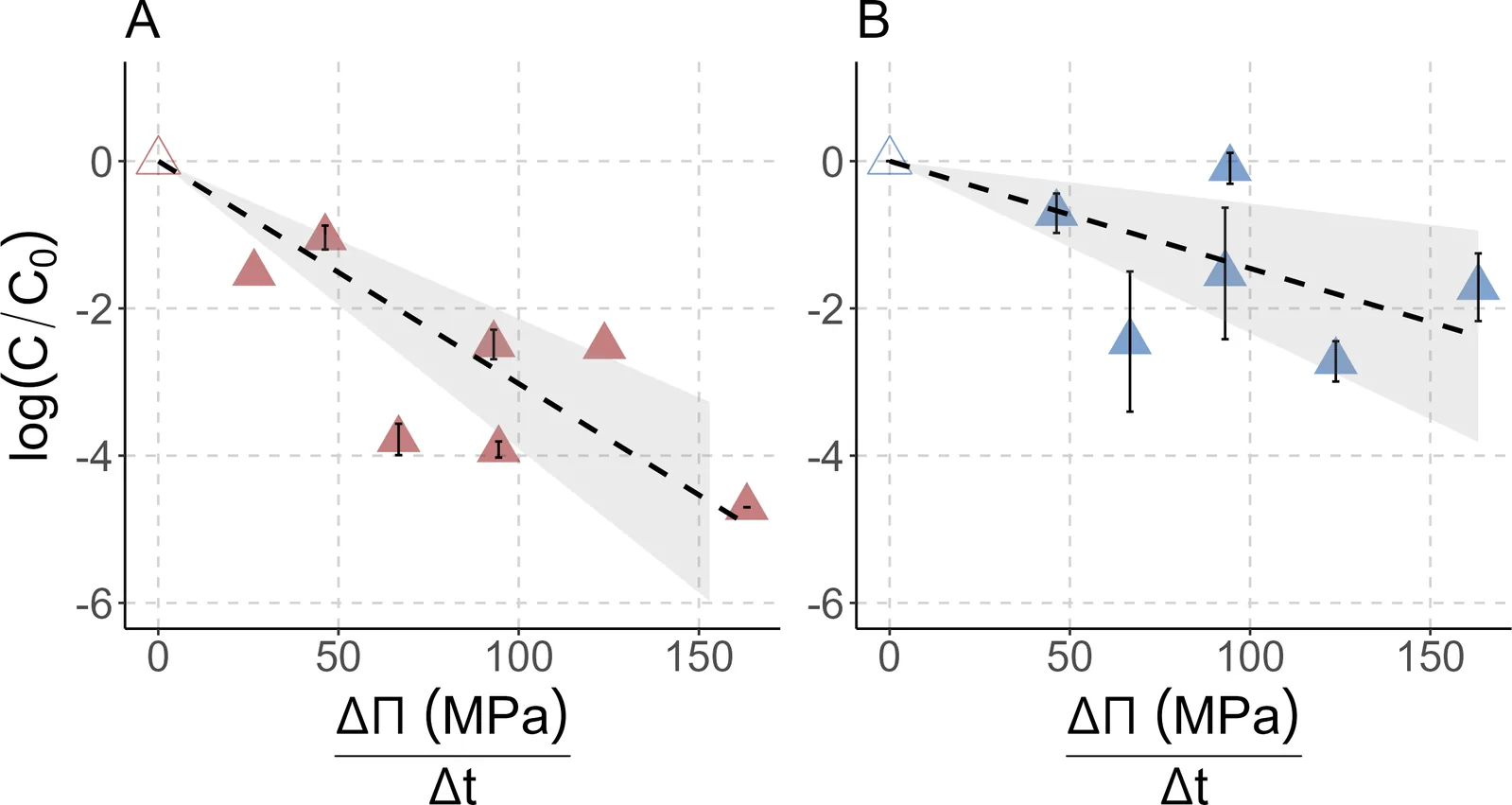
Correlation between the maximum change in osmotic pressure (ΔΠ/Δ*t*) measured 4 min before and 1 min after droplet equilibrium was reached and the inactivation [log_10_(*C*/*C*_0_)] of *E. coli* (**A**) and *S. epidermidis* (**B**) measured after droplet equilibrium. The error bars represent the standard deviation of all data points after droplet equilibrium. The empty triangle represents experiments performed in bulk 5.4 M NaCl (Table S2). The dashed lines show the fits obtained by linear regression, and the gray area indicates the associated 95% confidence intervals. The arrow on the *E. coli* graph shows that the point was placed at the quantification limit and could be lower (see Fig. 1A).

Given that inactivation only occurred while Π_droplet_ increased (Fig. 1), we determined that the loss of viable titer is associated with the rate of change in osmotic pressure (ΔΠ/Δ*t*). To this end, additional inactivation experiments were conducted and additional osmotic pressure changes modeled over an RH range from 40% to 80%, as well as in diluted and concentrated artificial saliva in order to determine the effect of different initial osmotic pressures on inactivation (Fig. S3, S5 to S7; Table S1). ΔΠ/Δ*t* at each RH was determined by ResAM, using a 5-min window during which droplet water activity reached equilibrium with the ambient RH (4 min before and 1 min after). The selected timescale also falls within the period over which *E. coli* cannot yet mount a full osmoadaptive response, which typically requires tens of minutes. Correlations were constructed across all artificial-saliva droplet experiments, relating the rate of osmotic pressure change as water activity approached equilibrium with the ambient RH to the corresponding reduction in bacterial titer. As shown in Table 1 and Fig. 2, the decrease in viable titer was log-linearly related to ΔΠ/Δ*t* for both bacteria.

**TABLE 1:** Log-linear regression coefficients for the relationship log_10_(*C*/*C*_0_) *=* β*(*ΔΠ/Δ*t)^a^*

Bacterium	β	*R*
*E. coli*	−0.030	0.95
*S. epidermidis*	−0.011	0.86

^*a*^ β is the slope associated with osmotic pressure change rate (MPa·5 min^−1^). *R *is the Pearson correlation coefficient calculated between the modeled ΔΠ/Δ*t *and the experimentally measured log_10_(*C*/*C*_0_) across all artificial-saliva droplet conditions included in the regression; log_10_(*C*/*C*_0_) refers to the averaged bacterial titers measured after equilibrium had been reached.

To assess whether this relationship reflects a general physical mechanism rather than a medium-specific effect, independent droplet experiments were performed in phosphate-buffered saline (PBS) at 60% RH for *E. coli* and 40% RH for *S. epidermidis*. Using the *Δ*Π/Δ*t–inactivation* relationships derived from artificial saliva (Fig. 2), predicted post-droplet equilibrium titers in PBS were calculated via ResAM and compared with experimentally measured values. PBS was chosen because it is chemically distinct and simpler than artificial saliva, allowing us to test whether the rate of osmotic pressure change alone can predict bacterial survival.

**Fig 3:**
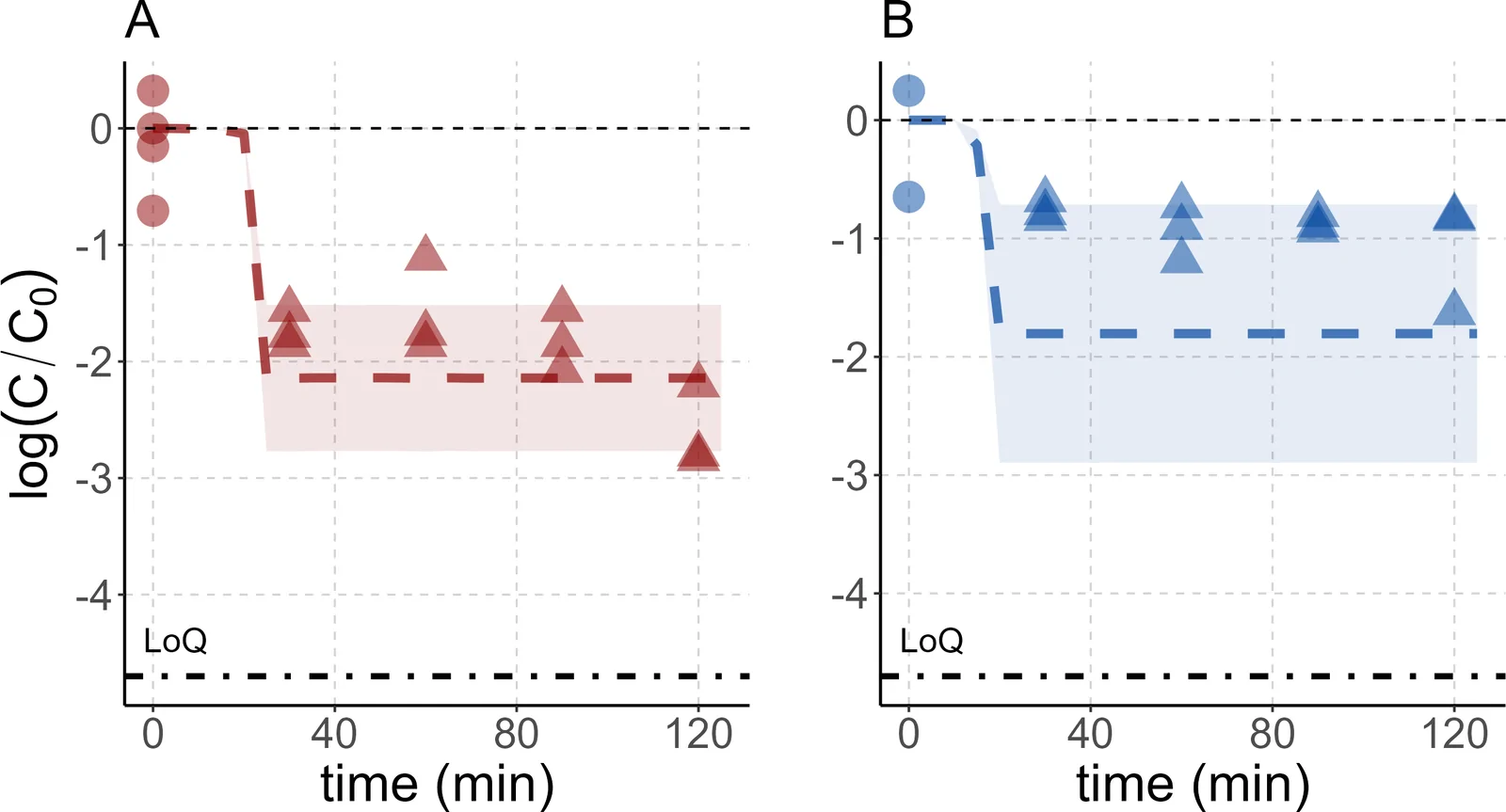
Bacterial inactivation [log_10_(*C*/*C*_0_)] of *E. coli* at 60% RH in PBS (**A**) and of *S. epidermidis* at 40% RH in PBS (**B**). Dashed lines represent model predictions. Data points indicate experimental measurements for *E. coli* (red) and *S. epidermidis* (blue), whereby circles represent liquid droplets and triangles represent effloresced droplets. The shaded regions represent the 95% confidence interval. Predictions were performed based on simulations of ΔΠ/Δ*t* in ResAM, combined with the ΔΠ/Δ*t*–inactivation relationships from artificial saliva in Fig. 2.

As shown in Fig. 3, predicted titers for both organisms closely tracked the experimental measurements in PBS. Model agreement with post-droplet equilibrium data were evaluated by quantifying the fraction of observations falling within the 95% confidence interval and by comparing observed and predicted values using paired Wilcoxon signed-rank tests on the effloresced droplet data points. For *E. coli*, 75% of observations fell within the confidence interval, and observed values did not differ significantly from predictions (*P* = 0.20), indicating good agreement. For *S. epidermidis*, 89% of observations fell within the confidence interval, but observed values differed significantly from predictions (*P* = 0.0039), indicating a systematic overestimation of inactivation despite the model capturing most of the observed variability.

### Rate of osmotic pressure change governs bacterial inactivation under decreasing RH

Having established that bacterial inactivation scales with the rate of osmotic pressure change during droplet drying, the inactivation dynamics of *E. coli* under decreasing RH conditions were next investigated. Because osmotic pressure increases as droplets dry but rises most sharply at efflorescence, varying the rate of RH decrease alters the osmotic pressure trajectory and thereby controls both the timing and magnitude of this critical osmotic shock.

Two droplet experiments were performed: one with a rapid decrease in RH from 90% to 30% and one with a gradual decrease (Fig. S8). Droplets effloresced under both conditions, and efflorescence also triggered the largest change in osmotic pressure. By comparing osmotic pressure, efflorescence timing, and post-efflorescence titers, it was assessed whether bacterial inactivation depends primarily on the abrupt osmotic pressure jump at efflorescence or on the slower osmotic pressure increase during drying.

**Fig 4:**
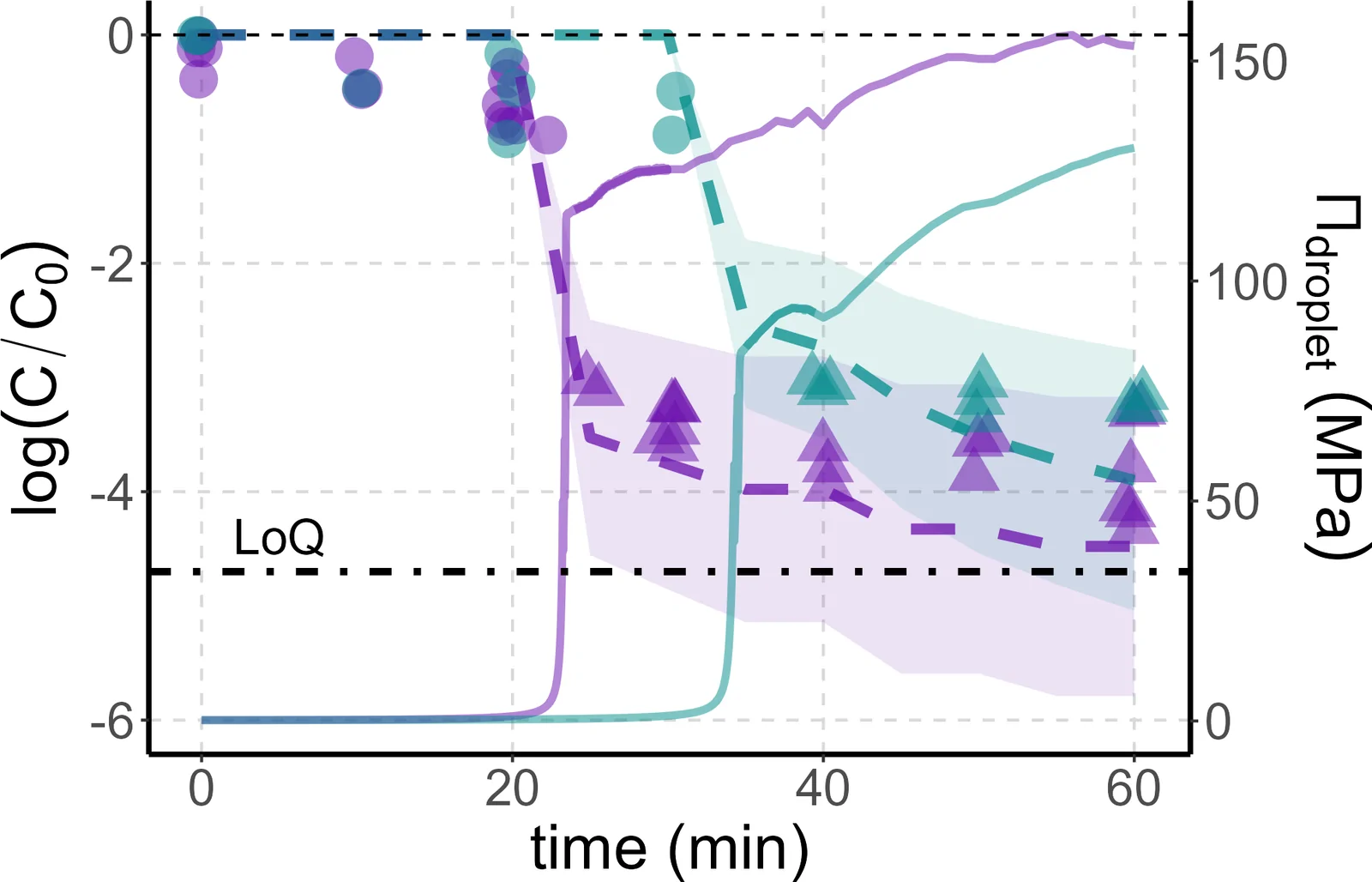
Fast relative humidity decrease (purple) and slow relative humidity (teal) with corresponding *E. coli* inactivation (circles represent liquid droplets and triangles represent effloresced droplets). The dashed line represents modeled *E. coli* from the model in Fig. 2. The solid line represents modeled osmotic pressure in the two experiments from ResAM. The shaded regions are the 95% confidence intervals for the linear model.

Figure 4 shows that *E. coli* viability is governed by the rapid osmotic pressure increase immediately before efflorescence. Although osmotic pressure rises progressively even after efflorescence, the decay in *E. coli* titer is only minor, indicating that this gradual increase is not sufficient to impose strong osmotic shock on the bacteria.

In the rapid drying experiment (purple), efflorescence occurred at about 23 min, corresponding to a ΔΠ/Δ*t* of 118 MPa. In the slow drying experiment (teal), efflorescence occurred at about 34 min, corresponding to a ΔΠ/Δ*t* of 85.9 MPa. Inactivation was greater under rapid drying (3.8 log_10_) than under slow drying (3.2 log_10_), consistent with efflorescence occurring at a lower RH and producing a larger osmotic pressure jump than in the slower drying case. Using the ΔΠ/Δ*t–*inactivation relationship in Fig. 2, the fast experiment has a predicted decay of 3.6 log_10_ while the slow experiment has a predicted decay of 2.8 log_10_.

To assess model agreement with the post-efflorescence data, the fraction of observed points falling within the 95% confidence interval of the model predictions was quantified, and observed and predicted values were compared using paired Wilcoxon signed-rank tests. Under the fast-drying condition, 100% of post-efflorescence observations fell within the model confidence interval, and observed values did differ significantly from predictions (*P =* 1.91 × 10^−6^) indicating systematic overestimations of the decay. Under the slow-drying condition, all observations fell within the confidence interval, and observed values did not differ significantly from model predictions (*P =* 0.15), indicating good model agreement.

## DISCUSSION

A quantitative relationship was established between the rate of change in osmotic pressure and the loss of viability of *E. coli* and *S. epidermidis* in artificial saliva droplets. Importantly, combining physicochemical modeling with the empirically derived ΔΠ/Δ*t*–inactivation relationship enabled prediction of bacterial inactivation during droplet drying. The results indicate that bacterial survival is governed not by the final osmotic pressure reached, but by the rate at which osmotic pressure increases as droplet water activity approaches equilibrium with the ambient RH, potentially exceeding bacterial adaptive capacity. Because this rate of change is strongly controlled by ambient RH, changes in indoor humidity can substantially alter microbial survival during droplet drying. By resolving the timing and magnitude of these transient osmotic changes, the model provides a mechanistic framework linking droplet drying dynamics to the observed loss of bacterial viability.

While both organisms exhibit increased inactivation with increasing rate of osmotic pressure change in droplets, *E. coli* is more susceptible to osmotic pressure changes compared to *S. epidermidis*. Inactivation was modest for both organisms at high relative humidity (70% RH), but diverged under typical indoor conditions: at 50% RH*, E. coli* exhibited ~3.8 log_10_ inactivation compared to ~1.5 log_10_ for *S. epidermidis*, with this disparity increasing further under drier conditions. This finding is consistent with prior reports on the relative sensitivities of these two organisms to desiccation and osmotic stress and implies that RH control is a more effective tool for inactivating gram-negative than gram-positive bacteria in IRPs (9). The stronger susceptibility of *E. coli* relative to *S. epidermidis* supports the broader paradigm that gram-negative bacteria are less tolerant of rapid changes in osmotic pressure than gram-positive organisms (13).

### Comparison to prior research

Contextualizing these findings within the existing literature provides insight into the mechanisms governing bacterial survival during droplet drying. Building on previous studies examining bacterial responses to osmotic and evaporative stress, the present work quantitatively resolves the rapid osmotic transitions occurring as droplet water activity approaches equilibrium with the ambient RH. Poirier et al. investigated *E. coli* responses to controlled hyperosmotic shocks by modulating water activity with glycerol and showed that viability depends strongly on the rate of osmotic change, with an optimal window in which cells can adapt (7). Although the magnitude of osmotic pressure changes imposed in their system was comparable to those modeled in our droplets, their observed survival of *E. coli* was substantially higher. This likely reflects the protective effects of glycerol and other organic osmolytes, which can reduce membrane damage in bacteria (22). Their results highlight that *E. coli* can tolerate gradual osmotic increases but not rapid ones, supporting our conclusion that rapid increases in osmotic pressure during droplet drying can exceed the physiological tolerance of gram-negative bacteria.

In addition to osmotic shock, other studies have identified oxidative stress as a potentially important inactivation mechanism in evaporating droplets. Liang et al. examined *E. coli* inactivation in artificial saliva droplets across RH gradients and separated the contributions of osmotic stress and thiocyanate (SCN^−^) toxicity (6). They found that osmotic stress dominated at low RH, while chemical toxicity became more influential as RH increased. Although their artificial saliva formulation differed from ours, their observation that faster evaporation intensifies osmotic stress complements our mechanistic interpretation. Our modeling adds to their framework by quantifying the rapid osmotic pressure increase as droplet water activity approaches equilibrium with the ambient RH, a transient change not captured in their analysis that directly corresponds to the period of maximum inactivation in our experiments.

Other work has proposed oxidative stress as a dominant inactivation mechanism in small, levitated aerosol droplets (20, 23). These aerosol studies use micrometer-scale droplets with high surface-to-volume ratios, which enhance interactions with reactive oxygen species (ROS) and produce stress pathways distinct from those in sessile droplets. This contrast helps explain why oxidative stress appears less relevant in our system: in addition to having possibly less ROS in our experiments, the lower surface-to-volume ratio of sessile droplets slows ROS accumulation, whereas water still equilibrates rapidly after efflorescence and generates a brief but extreme hyperosmotic shock which these aerosol studies cannot reproduce. This comparison underscores that dominant inactivation mechanisms shift with droplet size and evaporation dynamics (20).

Taken together, these studies contextualize our results by showing that while bacteria can withstand gradual osmotic changes or experience additional stresses in aerosols, the rapid increase in osmotic pressure during droplet drying can impose a level of stress that neither *E. coli* nor *S. epidermidis* can physiologically accommodate.

### Comparison to respiratory pathogens

Although *E. coli* and *S. epidermidis* serve as models in this study, pathogenic gram-positive respiratory bacteria often show strong desiccation tolerance: *Staphylococcus aureus* dried in PBS on polystyrene or stainless steel at 20–25% RH, ~22°C, exhibited <1 log_10_ loss after 7 days and only 1.5–2 log_10_ loss after 14–21 days (24). Similarly, *Streptococcus pneumoniae* dried from Todd-Hewitt broth with yeast onto plastic surfaces at 20–30% RH, 23–25°C, showed <1 log_10_ reduction after 24 h, with viable cells recovered after 4 days and 20–30% survival depending on serotype, indicating that gram-positive respiratory pathogens generally retain substantial viability under low-RH drying conditions (25). Based on their shared gram-positive physiology, many respiratory pathogens would be expected to display comparable resilience to our *S. epidermidis* model under droplet-drying conditions.

Several nontuberculous mycobacteria, which are taxonomically gram-positive Actinobacteria, exhibit substantial desiccation tolerance. Water-acclimated *Mycobacterium avium* and *M. chimaera* dried on filter paper at approximately 40% RH and 25°C retained 28% and 34% viability after 21 days, with one *M. chimaera* strain showing 100% survival over the same period (26). When dried as biofilms on stainless steel under the same conditions, *M. intracellulare* showed greater than 100% survival after 42 days, while *M. avium* and *M. abscessus* retained 18% and 14% survival, and *M. chelonae* survived up to 21 days. These results demonstrate that several respiratory-relevant bacteria with thick, complex cell envelopes can remain viable for extended periods at low relative humidity. Within this broader context, *E. coli* represents a highly sensitive, low-survival case, whereas *S. epidermidis* provides a more appropriate benchmark for the desiccation tolerance expected of clinically relevant respiratory bacteria in drying droplets.

In contrast to bacteria, previous work on influenza A virus in similar drying droplets reported continued inactivation after efflorescence, with a transition to slower, first-order decay rather than a plateau (3). This difference reflects distinct inactivation mechanisms. For influenza virus, inactivation is driven by sustained exposure to high salt concentrations and associated damage to virion structure, allowing decay to continue after equilibration. In contrast, bacterial inactivation in this study is confined to the brief period of rapid osmotic pressure increase as droplet water activity approaches equilibrium with the ambient RH, after which viability remains stable. This suggests that bacterial inactivation is driven by acute osmotic shock rather than prolonged exposure to elevated salinity.

### Implications for airborne IRPs

This study focuses on sessile droplets, though bacterial inactivation in airborne IRPs is likely a larger point of concern. It is therefore important to relate the findings from this work to bacterial inactivation in smaller, airborne IRPs. Small IRPs (<10–20 µm) evaporate extremely rapidly, cool significantly, and have much higher surface-to-volume ratios than the millimeter-scale droplets used here (27). These features accelerate solute concentration and also promote accumulation of reactive oxygen species, producing stress pathways likely not relevant in larger droplets (22). Importantly, aerosols also undergo rapid pH changes during evaporation, driven by volatilization of CO_2_, partitioning of volatile acids and bases, and concentration of buffering species (28, 29). Consequently, while aerosol inactivation reflects the combined influence of osmotic shock, oxidative stress, and rapid pH shifts, the much shorter evaporation timescales in airborne IRPs amplify the rate of osmotic pressure change to an even greater degree, suggesting that osmotic shock remains a key driver of bacterial loss in this regime (28).

### Limitations

This study has several limitations. Artificial saliva was used as the respiratory matrix, but mucin was excluded to enable more accurate modeling of osmotic pressure. Although mucin can alter droplet evaporation dynamics, previous work suggests it does not substantially affect bacterial survival (23). Nevertheless, droplet drying dynamics in this study may differ from those of real saliva. Another study by Longest et al. shows that using pooled human saliva results in a greater rate of inactivation of Influenza A virus, likely due to the presence of additional chemical constituents, including proteins, enzymes, and reactive species, that are not present in artificial saliva (30).

Second, the bacterial species used in this study, *E. coli* and *S. epidermidis*, serve as model organisms rather than true respiratory pathogens. Although they represent key structural differences between gram-negative and gram-positive bacteria, clinically relevant respiratory pathogens may possess distinct survival strategies, surface characteristics, and stress-response kinetics. Therefore, extrapolation of these findings to pathogenic species should be made with caution until similar experiments are conducted directly on those organisms.

Third, the experiments were performed using 1 μL droplets, which are larger than most respiratory droplets generated during breathing, speaking, or coughing. Because droplet size strongly influences evaporation dynamics, solute concentration rates, and the timing of efflorescence, smaller droplets may experience different osmotic pressure trajectories than those observed here. Future work should investigate whether similar osmotic shock dynamics occur in droplets more representative of airborne respiratory particles.

Additionally, the ΔΠ/Δ*t–*inactivation relationship used to relate bacterial inactivation to the rate of osmotic pressure change (Fig. 2) was constrained to a 5-min time window around droplet equilibrium. This limited the range of osmotic pressure changes captured by the model. The constraint was particularly relevant for 10× artificial saliva, where osmotic pressure increased more gradually and therefore reached a lower value within the 5-min window than in 1× or 0.1× artificial saliva (Fig. 2). Extending the observation window to 10 minutes would likely reduce these differences, as the full change in osmotic pressure would be more completely captured over that longer timeframe (see Fig. S5 for the evolution of osmotic pressure of a 10× artificial saliva droplet).

The physicochemical simulations were also subject to limitations associated with the ResAM framework. In particular, the model relies on assumptions regarding droplet composition, thermodynamic equilibrium, and homogeneous solute distribution during evaporation. These simplifications may not fully capture microscale concentration gradients, phase transitions, or spatial heterogeneity that can arise within drying respiratory droplets. Additional assumptions and parameter uncertainties associated with the model are summarized in the Supplemental material.

Taken together, this droplet-based system offers a controlled framework for quantifying osmotic shock and its effects on bacterial survival. However, it does not fully replicate the complex physicochemical environment of airborne respiratory aerosols. Future work extending this approach to airborne infectious respiratory particles will be necessary to capture the additional stress factors that influence microbial viability under real-world transmission conditions.

## MATERIALS AND METHODS

### Organism propagation, preparation, and enumeration

Detailed methods for organism propagation, preparation, and enumeration of *E. coli* (DSM-5695) and *S. epidermidis* (DSM-1798) are provided in the Supplemental material (Method S1).

### DNA extractions and quantification by digital PCR (dPCR)

Detailed protocols for DNA extraction and dPCR quantification are provided in the Supplemental material (Method S2; Tables S3 to S5).

### Droplet experiments

Droplet experiments were performed in an environmental chamber (Electro-tech Systems 5332) with controls for relative humidity and temperature. Prior to the experiment and during bacterial resuspension, the chamber was set to the desired relative humidity and temperature. Once the chamber was ready, 1 μL droplets of artificial saliva (Pickering Laboratories) or phosphate-buffered saline (PBS; Gibco) containing 10^8^ colony-forming units per mL of *E. coli* or *S. epidermidis* were deposited into a 96-well plate with non-binding surfaces (Greiner Bio-One, 655901), with one droplet per well. Droplets were left to dry over the course of 60 min for 30% (only for *E. coli*) and 120 min for other relative humidities and were periodically collected at each time point, with three droplets deposited per time point. The first three deposited droplets were collected immediately and were considered the *t*_0_ time point.

Droplets were collected by depositing 300 μL of PBS into the well. The droplets were then resuspended by pipetting up and down with a 200 μL pipette 10 times, scratching the bottom of the well with the pipette tip 5 times, and then repeating those two steps. Each sample was aliquoted into two 150-μL samples, one placed into a separate 96-well plate (Greiner) for an infectivity assay and the other into an Eppendorf tube for analysis by digital PCR. Droplets were visually inspected to determine whether crystallization had occurred. In each experiment, a control was included by sampling 1 μL of the experimental solution directly from the Eppendorf at both the start and conclusion of the experiment and kept inside the chamber to maintain the same conditions as the droplet experiment. See Table S2 for experimental parameters.

Additional droplet experiments were conducted in polyethylene glycol 200 (Carl Roth). The corresponding details are given in the Supplemental material (Method S3).

Inactivation experiments at constant saturated NaCl were conducted in bulk solutions. See the Supplemental material (Method S4) for details.

### Droplet experiments under changing relative humidity

Detailed experimental procedures for changing relative humidity experiments are described in the Supplemental material (Method S5).

### Osmotic pressure modeling

Each droplet was modeled using the respiratory aerosol model (ResAM), a spherical shell diffusion model that simulates the evolution of physicochemical conditions within drying respiratory droplets. The model resolves radial concentration gradients and water activity across concentric shells as evaporation proceeds. The model was initialized using the measured environmental conditions inside the chamber, including relative humidity and temperature together with the initial chemical droplet composition and the experimentally measured droplet size evolution obtained from video recordings.

The original version of ResAM is fully described in Luo et al. (28), which was adapted for virus-containing droplets in Schaub et al. (3). For droplets containing *E. coli* and *S. epidermidis*, the model was further adapted to account for bacteria changing the droplet morphology and increasing the rate of evaporation (31). More details for the model adaptations, inputs, and parameters are described in the supplemental material (Method S6; Fig. S9 and S10; Tables S6 and S7). Water activity values simulated by ResAM were used to calculate the osmotic pressure within each droplet shell according to the van’t Hoff equation (8):


$$\Pi =\left(-RT/V_{w}\right)\ln\left(a_{w}\right)$$

where Π is the osmotic pressure in MPa, *R* is the universal gas constant (8.314 J mol^−1^ K^−1^), *T* is temperature in Kelvin (298 K), *V*_*w*_ is the molar volume of water at 298 K (18.015 × 10^−6^ m^3^), and *a*_*w*_ is the water activity output of ResAM. This formulation defines osmotic pressure relative to pure water (*a*_*w*_ = 1). After the droplet reaches equilibrium, the calculated osmotic pressure refers to the residual liquid phase remaining in equilibrium with the crystalline salts rather than to the solid crystal lattice itself. The osmotic pressure was averaged across shells to generate the line plot visualizations. ResAM simulations were executed on a high-performance computing cluster, and the resulting shell-resolved outputs were processed and analyzed in R (version 2023.09.1).

### Determination of ΔΠ/Δ*t*–inactivation relationship

To quantify the rate of osmotic pressure change, the time of hyperosmotic shock was first identified for each droplet. A 5-min window spanning this event (4 min before and 1 min after droplet equilibration) was then extracted. This interval captures the rapid change in osmotic pressure as droplet water activity approaches equilibrium with the ambient RH while remaining short relative to the droplet sampling timescale. Within this window, the change in osmotic pressure (ΔΠ/Δ*t*) was calculated from the ResAM output and related to the experimentally observed inactivation using linear regression. The selected timescale also falls within the period over which *E. coli* cannot yet mount an osmoadaptive response, which typically requires tens of minutes. We therefore assume that Π_bacteria_ does not change over the 5-min window considered; the change in turgor pressure *P*_turgor_ before and after droplet equilibrium, (Π_bacteria_ *−* Π_droplet, before_) − (Π_bacteria_ *−* Π_droplet*,* after_), then corresponds to ΔΠ_droplet_ *=* Π_drople*t,* after_ *−* Π_droplet, before_*,* here simply termed ΔΠ.

Post-equilibrium bacterial titers measured at a given RH in artificial saliva droplets were averaged and linked to the corresponding ΔΠ*.* The point at the origin represents the osmotic pressure difference between PBS, in which the bacteria were initially suspended, and a 5.4 M NaCl solution (see Methods S4 and S7).

### Prediction of bacterial titers

To predict bacterial titers in other matrices or under different RH conditions, ΔΠ/Δ*t* was calculated from the ResAM output by determining the slope over consecutive 5-min intervals. These values were then used together with the experimentally derived ΔΠ/Δ*t–*inactivation relationship to estimate bacterial decay at each interval. Cumulative bacterial inactivation was calculated by summing the predicted decay across successive intervals. The model assumes that inactivation is primarily associated with rapid changes in osmotic pressure during droplet drying; therefore, predicted bacterial titers remain constant once osmotic pressure reaches equilibrium unless additional osmotic changes occur. Inactivation was only applied when the change in osmotic pressure exceeded 5 MPa; otherwise, titers were assumed to remain unchanged.

### Statistical analysis

All statistical analyses were performed in R (version 2023.09.1). Linear regression was used to quantify relationships between osmotic pressure change and bacterial inactivation. Differences between predicted and observed titers were evaluated using paired Wilcoxon signed-rank tests. Efflorescence time distributions were fitted using the *fitdistrplus* package in R. Differences in bacterial survival between *E. coli* and *S. epidermidis* were evaluated using analysis of covariance (ANCOVA), with species, relative humidity, and their interaction included as fixed effects and sampling time included as a covariate. Pairwise comparisons of estimated marginal means were performed using the *emmeans* package. Significance was assessed at α = 0.05. Error bars represent the standard deviation of biological replicates.

## Data Availability

Experimental data are available in the public repository Zenodo under the following link: https://doi.org/10.5281/zenodo.21902064. All code is available at https://github.com/medinataylor/dropletpaper.

## SUPPLEMENTAL MATERIAL

Supplemental Material msphere.00594-26-s0001.docx Methods S1-S7, Fig. S1-S10, and Tables S1-S7.

ASM does not own the copyrights to Supplemental Material that may be linked to, or accessed through, an article. The authors have granted ASM a non-exclusive, world-wide license to publish the Supplemental Material files. Please contact the corresponding author directly for reuse.

## References

[1] World Health Organization. 2024. Global technical consultation report on proposed terminology for pathogens that transmit through the air. World Health Organization, Geneva, Switzerland.

[2] Lin K, Schulte CR, Marr LC. 2020. Survival of MS2 and Φ6 viruses in droplets as a function of relative humidity, pH, and salt, protein, and surfactant concentrations. PLoS One 15:e0243505. doi:10.1371/journal.pone.0243505 PMC7723248 33290421

[3] Schaub A, Luo B, David SC, Glas I, Klein LK, Costa LJ, Terrettaz C, Bluvshtein N, Motos G, Violaki K, Pohl MO, Hugentobler W, Nenes A, Stertz S, Krieger UK, Peter T, Kohn T. 2024. Salt supersaturation as an accelerator of influenza A virus inactivation in 1 μL droplets. Environ Sci Technol 58:18856–18869. doi:10.1021/acs.est.4c04734 39392017

[4] Jarvis MC. 2021. Drying of virus-containing particles: modelling effects of droplet origin and composition. J Environ Health Sci Eng 19:1987–1996. doi:10.1007/s40201-021-00750-6 PMC8569499 34754455

[5] Davis RD, Lance S, Gordon JA, Ushijima SB, Tolbert MA. 2015. Contact efflorescence as a pathway for crystallization of atmospherically relevant particles. Proc Natl Acad Sci USA 112:15815–15820. doi:10.1073/pnas.1522860113 PMC4702978 26668396

[6] Liang Z, Chan WL, Tian X, Lai ACK, Lee PKH, Chan CK. 2022. Inactivation of *Escherichia coli* in droplets at different ambient relative humidities: effects of phase transition, solute and cell concentrations. Atmos Environ 280:119066. doi:10.1016/j.atmosenv.2022.119066

[7] Poirier I, Maréchal PA, Gervais P. 1997. Effects of the kinetics of water potential variation on bacteria viability. J Appl Microbiol 82:101–106. doi:10.1111/j.1365-2672.1997.tb03303.x 9113880

[8] Zheng S, Li Y, Shao Y, Li L, Song F. 2024. Osmotic pressure and its biological implications. Int J Mol Sci 25:3310. doi:10.3390/ijms25063310 PMC10970305 38542282

[9] Wennerström H, Oliveberg M. 2022. On the osmotic pressure of cells. QRB Discov 3:e12. doi:10.1017/qrd.2022.3 PMC10392628 37529285

[10] Arnoldi M, Fritz M, Bäuerlein E, Radmacher M, Sackmann E, Boulbitch A. 2000. Bacterial turgor pressure can be measured by atomic force microscopy. Phys Rev E 62:1034–1044. doi:10.1103/PhysRevE.62.1034 11088560

[11] Smith BL, King MD. 2023. Quiescence of *Escherichia coli* aerosols to survive mechanical stress during high-velocity collection. Microorganisms 11:1806. doi:10.3390/microorganisms11030647 PMC10058004 36985220

[12] Pilizota T, Shaevitz JW. 2012. Fast, multiphase volume adaptation to hyperosmotic shock by *Escherichia coli*. PLoS One 7:e35205. doi:10.1371/journal.pone.0035205 PMC3325977 22514721

[13] Wood JM. 1999. Osmosensing by bacteria: signals and membrane-based sensors. Microbiol Mol Biol Rev 63:230–262. doi:10.1128/MMBR.63.1.230-262.1999 PMC98963 10066837

[14] Ferreira RBR, Ferreira MCS, Glatthardt T, Silvério MP, Chamon RC, Salgueiro VC, Guimarães LC, Alves ES, Dos Santos KRN. 2019. Osmotic stress induces biofilm production by *Staphylococcus epidermidis* isolates from neonates. Diagn Microbiol Infect Dis 94:337–341. doi:10.1016/j.diagmicrobio.2019.02.009 30885396

[15] Sleator RD, Hill C. 2002. Bacterial osmoadaptation: the role of osmolytes in bacterial stress and virulence. FEMS Microbiol Rev 26:49–71. doi:10.1111/j.1574-6976.2002.tb00598.x 12007642

[16] Xie X, Li Y, Zhang T, Fang HHP. 2006. Bacterial survival in evaporating deposited droplets on a teflon-coated surface. Appl Microbiol Biotechnol 73:703–712. doi:10.1007/s00253-006-0492-5 PMC7080038 17053902

[17] Agharkar AN, Hajra D, Roy D, Jaiswal V, Kabi P, Chakravortty D, Basu S. 2024. Evaporation of bacteria-laden surrogate respiratory fluid droplets: on a hydrophilic substrate versus contact-free environment confers differential bacterial infectivity. Phys Fluids 36:031912. doi:10.1063/5.0196219

[18] Lin K, Marr LC. 2020. Humidity-dependent decay of viruses, but not bacteria, in aerosols and droplets follows disinfection kinetics. Environ Sci Technol 54:1024–1032. doi:10.1021/acs.est.9b04959 31886650

[19] Chiera S, Bosco F, Mollea C, Piscitello A, Sethi R, Nollo G, Caola I, Tessarolo F. 2023. *Staphylococcus epidermidis* is a safer surrogate of *Staphylococcus aureus* in testing bacterial filtration efficiency of face masks. Sci Rep 13:21807. doi:10.1038/s41598-023-49005-4 PMC10710463 38071253

[20] Otero-Fernandez M, Thomas RJ, Oswin H, Alexander R, Haddrell AE, Reid JP. 2024. Inactivation mechanisms of *Escherichia coli* in simulants of respiratory and environmental aerosol droplets. Atmosphere 15:511. doi:10.3390/atmos15040511

[21] Krieger UK, Siegrist F, Marcolli C, Emanuelsson EU, Gøbel FM, Bilde M, Marsh A, Reid JP, Huisman AJ, Riipinen I, Hyttinen N, Myllys N, Kurtén T, Bannan T, Percival CJ, Topping D. 2018. A reference data set for validating vapor pressure measurement techniques: homologous series of polyethylene glycols. Atmos Meas Tech 11:49–63. doi:10.5194/amt-11-49-2018

[22] Kempf B, Bremer E. 1998. Uptake and synthesis of compatible solutes as microbial stress responses to high-osmolality environments. Arch Microbiol 170:319–330. doi:10.1007/s002030050649 9818351

[23] Oswin HP, Haddrell AE, Hughes C, Otero-Fernandez M, Thomas RJ, Reid JP. 2023. Oxidative stress contributes to bacterial airborne loss of viability. Microbiol Spectr 11:e03347-22. doi:10.1128/spectrum.03347-22 PMC10101003 36912675

[24] Chaibenjawong P, Foster SJ. 2011. Desiccation tolerance in *Staphylococcus aureus*. Arch Microbiol 193:125–135. doi:10.1007/s00203-010-0653-x 21088825

[25] Walsh RL, Camilli A. 2011. *Streptococcus pneumoniae* is desiccation tolerant and infectious upon rehydration. mBio 2:e00092-11. doi:10.1128/mBio.00092-11 PMC3101785 21610120

[26] Falkinham JO III, Williams MD. 2022. Desiccation tolerance of *Mycobacterium avium*, *Mycobacterium intracellulare*, *Mycobacterium chimaera*, *Mycobacterium abscessus* and *Mycobacterium chelonae*. Pathogens 11:463. doi:10.3390/pathogens11040463 PMC9028797 35456138

[27] Jarvis MC. 2020. Aerosol transmission of SARS-CoV-2: physical principles and implications. Front Public Health 8:590041. doi:10.3389/fpubh.2020.590041 PMC7719704 33330334

[28] Luo B, Schaub A, Glas I, Klein LK, David SC, Bluvshtein N, Violaki K, Motos G, Pohl MO, Hugentobler W, Nenes A, Krieger UK, Stertz S, Peter T, Kohn T. 2023. Expiratory aerosol pH: the overlooked driver of airborne virus inactivation. Environ Sci Technol 57:486–497. doi:10.1021/acs.est.2c05777 PMC9835828 36537693

[29] Haddrell AE, Otero-Fernandez M, Oswin H, Cogan TA, Bazire J, Tian J, Alexander RW, Mann JFS, Hill DJ, Finn AHR, Davidson AD, Reid JP. 2023. Differences in airborne stability of SARS-CoV-2 variants of concern is impacted by alkalinity of surrogates of respiratory aerosol. J R Soc Interface 20:20230062. doi:10.1098/rsif.2023.0062 PMC10282576 37340783

[30] Longest AK, Srivastava S, Mazzola FA, Smeltz RE, Parks JL, Klein LK, Duggal NK, Vikesland PJ, Marr LC. 2026. Influence of carbon dioxide and pH on influenza virus in sessile saliva droplets. Environ Sci Technol 60:7274–7282. doi:10.1021/acs.est.5c12672 PMC12980841 41744156

[31] David SC, Schaub A, Terrettaz C, Motos G, Costa LJ, Nolan DS, Augugliaro M, Wynn HK, Glas I, Pohl MO, Klein LK, Luo B, Bluvshtein N, Violaki K, Hugentobler W, Krieger UK, Peter T, Stertz S, Nenes A, Kohn T. 2024. Stability of influenza A virus in droplets and aerosols is heightened by the presence of commensal respiratory bacteria. J Virol 98:e00409-24. doi:10.1128/jvi.00409-24 PMC11264603 38869284

