# Supplementary material for: Rate of osmotic pressure change in drying saliva microdroplets drives inactivation of model respiratory bacteria
Source: mSphere. 2026 Oct 7;11(10):e00594-26. doi: 10.1128/msphere.00594-26 (PMC13711787; doi:10.1128/msphere.00594-26)
Supplement: Supplementary file 1 [file msphere.00594-26-s0001.docx]

**Supporting information for:**

Rate of osmotic pressure change in drying saliva microdroplets drives inactivation of model respiratory bacteria

Taylor Medina^a^, Beiping Luo^b^, Thomas Peter^b^, Htet Kyi Wynn^a^, Tamar Kohn^a#^

^a^Laboratory of Environmental Virology, School of Architecture, Civil & Environmental Engineering, École Fédérale Polytechnique de Lausanne, Lausanne, Switzerland

^b^Institute for Atmospheric and Climate Science, ETH Zürich, Zürich, Switzerland

**This file includes:**

20 pages

Supporting Methods S1-S7

Figures S1 – S10

Tables S1 – S7

**Supplemental Methods**

**Method S1 - Organism Propagation and Purification**

Experiments were conducted using *Escherichia coli* (DSM-5695) and *Staphylococcus epidermidis* (DSM-1798). For *E. coli*, 10 mL Luria-Bertrani Broth (LB Broth) (Invitrogen 12795027) was added to a 15 mL falcon tube and 100 μL of *E. coli* stock was inoculated and then incubated at 37°C overnight with shaking at 200 rpm. Before each droplet experiment, the *E. coli* was vortexed and 1 mL was aliquoted into a 1.5 mL Eppendorf tube and centrifuged for four minutes at 10’000 x g. The supernatant was discarded and the pellet was resuspended in 1 mL of artificial saliva (Pickering Laboratories). For *S. epidermidis*, 10 mL of yeast-enhanced tryptic soy broth (TSB, Millipore 102512952 with 3g of yeast extract added per liter) was added to a 15 mL falcon tube and 100 μL of *S. epidermidis* was inoculated and then incubated in 37°C overnight. Resuspension into artificial saliva follows the same protocol as *E. coli*.

Live bacteria were quantified by plating 10 µL of 10-fold serial dilutions onto organism-specific agar media. *E. coli* was enumerated on LB agar (15g agar per liter), while *S. epidermidis* was enumerated on tryptic soy agar (TSA, Fluka Analytical 22091) supplemented with yeast extract (3g yeast per liter). Plates were incubated at 37 °C for 18–24 h, after which colonies were counted and concentrations were reported as colony-forming units (CFU) on the order of 10^8^ CFU/mL.

The limit of quantification (LoQ) was defined as the concentration corresponding to at least two detected colonies (2 × 10³ CFU/mL), corresponding to a maximum quantifiable reduction of 4.7 log₁₀ from an initial concentration of 10⁸ CFU/mL.

**Method S2 - DNA Extraction and Digital PCR Quantification**

DNA from *E. coli* and *S. epidermidis* was independently extracted using the Maxwell RSC Instrument (Promega) together with the Maxwell RSC Whole Blood DNA Kit (AS1400) according to the manufacturer’s instructions. DNA was eluted in 60 μL of supplied elution buffer and stored at −20 °C until quantification.

Extracted DNA was quantified using digital PCR (dPCR) with either the QIAcuity 16S *E. coli* Kit (Qiagen, cat. no. 18000-16) or the QIAcuity 16S *S. aureus* Kit (Qiagen, cat. no. 250207). All reactions were loaded into QIAcuity Nanoplate 8.5k 96-well plates (Qiagen, cat. no. 250021) and run on the QIAcuity One Digital PCR System (Qiagen, 5-plex device, cat. no. 911255).

The thermal cycling protocol consisted of enzyme activation at 95 °C for 2 min followed by 40 cycles of 95 °C for 15 s and 58 °C for 1 min. All dPCR runs included both positive controls (Qiagen, cat. no. 338135) and negative controls consisting of RNase-free water. To account for variations in recovery efficiency, CFU measurements were normalized to the number of genome copies detected at time 0.

**Method S3 – PEG 200 Droplet Experiment**

To determine whether bacterial inactivation is driven by the osmotic pressure change associated with droplet equilibration or by salt crystal formation during efflorescence, droplet experiments were performed using polyethylene glycol 200 (PEG-200) at 50% RH. As with all other droplet experiments, the environmental chamber was pre-equilibrated to 50% RH prior to droplet deposition, such that droplets were immediately exposed to the target relative humidity upon placement in the chamber. Unlike inorganic salts, PEG-200 remains liquid while spanning a wide range of water activities, allowing osmotic stress to be studied in the absence of crystallization.

The relationship between PEG-200 composition and water activity has been previously characterized and is described by Krieger et al., 2018^3^:

$$a_{w}=\left( 1-x \right)exp\left[ -0.5512\left( 1+\frac{0.55152}{2.74096}\frac{\left( 1-x \right)}{x} \right)^{-2} \right]$$

where (x) is the mole fraction of PEG-200.

A PEG-200 solution with an initial water activity of 0.99, corresponding to that of artificial saliva, was prepared using this relationship. The calculated composition was 9.14 wt% PEG-200, prepared by mixing 0.914 g PEG-200 with 9.086 g deionized water.

**Method S4 - Stability of Bacteria in Saturated Sodium Chloride Solution**

To evaluate the effect of salt exposure alone on bacterial survival, 20 μL of either *E. coli* or *S. epidermidis* suspended in PBS was added to 180 μL of saturated NaCl solution (5.4 M) in technical triplicate at room temperature. Samples were collected immediately (time 0) and after 24 h. As shown in Figure S3, *S. epidermidis* remained stable over the 24 h exposure period, whereas *E. coli* exhibited approximately 1-log inactivation.

**Method S5 - Relative Humidity Change Experiments**

To investigate the effect of changing relative humidity (RH) on droplet behavior, two RH-transition protocols were performed.

For the fast RH-decrease experiment, the environmental chamber was initially equilibrated to 90% RH. Following droplet deposition, the chamber setpoint was switched to 30% RH, allowing the humidity to decrease naturally over the course of the experiment. Droplets were sampled at 0, 10, 20, 30, 40, 50, and 60 min, with additional sampling points at 22.5 and 25 min to capture the efflorescence transition.

For the slow RH-decrease experiment, the chamber was likewise initialized at 90% RH; however, the RH setpoint was reduced stepwise by 10% every 10 min. For ResAM simulations, the RH input for the fast-change condition was taken as the average RH measured across the two replicate fast-change experiments. See Figure S8 for the relative humidity change plot.

**Method S6 - ResAM Model Adaptations**

The full ResAM model for airborne particles is described in Luo et al. (2023)^1^. The model was subsequently extended by Schaub et al. (2024)^2^ to account for droplets deposited on a substrate. In the present work, droplet flattening on a substrate was represented by incorporating an inert central core, the size of which was determined from experimental image analysis. The remaining liquid volume was assumed to be distributed as a hemisphere. To maintain geometric consistency, the measured image area was matched to the surface area of a hemisphere with a mean radius defined as the average of the inner and outer radii. The core size was treated as time-dependent and iteratively determined from the experimentally observed image area together with the calculated droplet volume.

Compared to the version presented by Luo et al. (2023), two major modifications were implemented:

1. **Multi-shell solidification**
   Solid formation was enabled in all shells of the droplet rather than being restricted to the center.
2. **Improved diffusion treatment**
   Experimentally realistic ion diffusion coefficients were incorporated for crystal growth calculations. This replaced the previous parameterized enhancement-factor approach used by Luo et al. (2023), which constrained crystallization to the droplet center.

In addition, the chemical system was substantially expanded to include a broader range of aqueous and solid-phase species.

**Chemical Species Included in the Model**

The model included water and dissociated ions (H₂O, H⁺, OH⁻); two generic organic species with different molar masses and molar volumes; cations (Na⁺, K⁺, Ca²⁺, Mg²⁺); anions (Cl⁻, NO₃⁻); carbon species (H₂CO₃, HCO₃⁻, CO₃²⁻); ammonia species (NH₃, NH₄⁺); acetic acid species (CH₃COOH, CH₃COO⁻); phosphate species (H₃PO₄, H₂PO₄⁻, HPO₄²⁻, PO₄³⁻); sulfate species (HSO₄⁻, SO₄²⁻); lactic acid species (CH₃CH(OH)COOH, CH₃CH(OH)COO⁻); carbonic anhydrase enzyme; and additional acid species (default: oxalic acid) together with associated ions up to triple charge.

**Gas-Phase Species**

The following gas-phase species were included in the model: H₂O, HCl, CO₂, HNO₃, NH₃, CH₃COOH, lactic acid, and an additional acid species (default: oxalic acid).

For this study, air chemistry interactions were simplified by assuming exchange with CO₂ only at a concentration of 400 ppm.

**Solid Phases Considered for Efflorescence**

The following crystalline solids were included in the efflorescence calculations: NaCl, KCl, CaCO₃, MgCO₃, CaHPO₄, NaH₂PO₄, Na₂HPO₄·7H₂O, NH₄HC₂O₄·0.5H₂O, (NH₄)₂C₂O₄, NaHC₂O₄, Na₂C₂O₄, and H₂C₂O₄·7H₂O.

Note that not all crystalline phases listed above were used in the present study. See Table S2 for what species were used in the model inputs.

**ResAM Model Inputs**

Artificial saliva composition was based on the formulation provided by Pickering Laboratories (Table S6). Molality values used as model inputs were calculated from the reported composition. For PBS simulations, molality and molarity were assumed to be approximately equivalent; therefore, the concentrations listed in Table S2 were used directly as model inputs. The initial droplet diameter used as input to ResAM was determined from videography measurements of the deposited droplets (see below) and was 7.82 × 10⁻² cm.

At 30% RH, ResAM calculations terminated following efflorescence. Consequently, osmotic pressure values beyond efflorescence were manually extended using the equation presented in the main text with a water activity value corresponding to equilibrium at 30% RH (*a_w_* = 0.3). The same approach was applied to the 50% RH simulations, where calculations terminated after 100 min and were extended to 120 min.

**Confirmation of droplet crystallization time in the presence of bacteria by videography**

ResAM calculates efflorescence times based on physicochemical information only. To determine if modeled efflorescence times also apply in the presence of bacteria, evaporation and crystallization behavior of droplets containing *E. coli* or *S. epidermidis* were monitored across multiple RH conditions by videography. Images were acquired using a Sony IMX477R camera connected to a Raspberry Pi 4 computer (Model B Rev. 1.4) powered by a Varta power bank. The camera was positioned 15 cm above the 96-well plate and captured one image every 16 s.

Droplet area and diameter were quantified using ImageJ (version 1.53t). A single well of the 96-well plate was used as the spatial reference for image calibration. Efflorescence times were determined by manually inspecting image sequences frame-by-frame and recording the onset and completion of crystallization events.

At 30% RH, *S. epidermidis* droplets effloresced earlier than *E. coli*, whereas both species exhibited similar efflorescence times at 50% RH (Figure S9, Table S7). At 70% RH, no crystallization occurred (Figure S10).

Consequently, ResAM was used to estimate efflorescence times across all RH conditions. Model-predicted values fell within the experimentally observed range for *E. coli* and were consistent with observations for *S. epidermidis* (Table S7).

**Method S7 - Bulk Osmotic Pressure Calculations**

The osmotic pressure of the bulk solutions was calculated using the ideal solution osmotic pressure formula, with a correction factor for nonideality (ϕ) for the saturated NaCl solution.

*Π = iϕCRT*

*Π* is the osmotic pressure, *i* is the Van’t Hoff Factor, *C* is the molar concentration, *R* is the universal gas constant, and *T* is the temperature. The empty triangle in Figure 2 was calculated using the difference of 5.4M NaCl and PBS, with a value of **33.5 MPa**. See Table S1 for osmotic pressure calculations.

**Supplementary Figures**

**Figure S1:** Zoomed-in osmotic pressure curve from Figure 1 for 1x Artificial Saliva at 50%.

**Figure S2:** Genome copies (GC) normalized to time 0 for Figure 1. *E. coli* droplets are in red and *S. epidermidis* droplets are in blue.

**Figure S3:** Stability of *E. coli* (red) and *S. epidermidis* (blue) in bulk 5.4M NaCl solution

**Figure S4:** *E. coli* (red) and *S. epidermidis* (blue) droplet experiment in PEG-200 (a_w,0_ = 0.99). Grey are the controls in bulk PEG-200 (a_w_ = 0.99).

**Figure S5:** 10x Artificial Saliva osmotic pressure curve zoomed in from 10-30 minutes.

**Figure S6:** *E. coli* time series for 10x Artificial Saliva at 50% (purple), 0.1x Artificial Saliva at 50% (green), 40% (orange), and 80% (black).

**Figure S7:** *S. epidermidis* time series 10x Artificial Saliva at 50% (purple), 0.1x Artificial Saliva at 50% (green), and 40% (orange)

**Figure S8:** Relative Humidity change for the fast RH change experiments (purple) and the slow relative humidity change (teal) corresponding to Figure 4 in the main text.

**Figure S9:** Panel A shows the distributions of droplet efflorescence times for *E. coli* at 30% RH (orange, n = 21) and 50% RH (green, n = 24), and panel B shows the corresponding Q–Q plots used to assess normality. Panel C shows the distributions of droplet efflorescence times for *S. epidermidis* at 30% RH (yellow, n = 48) and 50% RH (blue, n = 36), and panel D shows the corresponding Q–Q plots used to assess normality. Dashed vertical lines in the distribution plots indicate the model-predicted efflorescence times (13.5 min at 30% RH and 18.6 min at 50% RH).

**Figure S10:** The photo on the left shows a liquid droplet, the photo in the middle shows an effloresced droplet, and the photo on the right shows a droplet at 70% that does not have a crystallization.

**Supplementary Tables**

**Table S1:** Summary of RH and Temperature Conditions by RH (µ ± σ)

|  | ***E. coli*** | | ***S. epidermidis*** | |
| --- | --- | --- | --- | --- |
| **Media** | **RH** | **Temperature** ºC | **RH** | **Temperature** ºC |
| 1x AS | 31.22 ± 1.46 | 22.56 ± 0.11 | 30.96 ± 0.71 | 23.95 ± 0.86 |
|  | 42.56 ± 0.80 | 22.08 ± 0.16 | 42.95 ± 0.77 | 23.06 ± 0.76 |
|  | 51.00 ± 1.45 | 22.62 ± 0.11 | 50.72 ± 0.23 | 24.55 ± 0.43 |
|  | 69.81 ± 3.80 | 21.83 ± 0.85 | 70.43 ± 1.01 | 24.66 ± 0.46 |
|  | 84.43 ± 1.50 | 23.52 ± 0.34 |  | |
| 10x AS | 50.42 ± 4.43 | 21.09 ± 0.83 | 50.87 ± 0.72 | 24.85 ± 0.86 |
| 0.1x AS |  |  |  |  |
| PBS | 60.10 ± 1.20 | 22.85 ± 0.40 | 42.95 ± 0.77 | 23.06 ± 0.76 |

**Table S2:** Osmotic Pressure Calculations

| **Chemical** | **C (mol·L^-1^)** | ***i*** | **R (L·MPa·mol⁻¹·K⁻¹)** | **T (K)** | **ϕ** | **Π (MPa)** |
| --- | --- | --- | --- | --- | --- | --- |
| ***5.4M NaCl*** | | | | | | |
| NaCl | 5.4 | 2 | 0.008314 | 298 | 1.28^4^ | **34.3** |
| ***PBS*** | | | | | | |
| NaCl | 0.137 | 2 | 0.008314 | 298 | 1.00 | 0.679 |
| KCl | 0.00270 | 2 |  |  |  | 0.0134 |
| Na₂HPO₄ | 0.0100 | 3 |  |  |  | 0.0743 |
| KH₂PO₄ | 0.00180 | 2 |  |  |  | 0.00892 |
| Total PBS | | | | | | **0.775** |

**Table S3:** *E. coli* dPCR reaction mix

| ***E. coli* Assay** | **Reaction mix for 1 sample (1 μL)** |
| --- | --- |
| QIAcuity OneStep Advanced Probe – Mastermix (4x) | 3 |
| Microbial DNA Assay *E. coli* 20x (DMA00140-F) | 0.6 |
| GC enhancer | 1.5 |
| Ultrapure H_2_O | 2.9 |
| Sample | 4 |
| Final volume | 12 |

**Table S4:** *S. epidermidis* dPCR Reaction Mix

| ***S. epidermidis* Assay** | **Reaction mix for 1 sample (1 μL)** |
| --- | --- |
| QIAcuity OneStep Advanced Probe – Mastermix (4x) | 3 |
| Microbial DNA Assay *S. epidermidis* 20x (DMA00304-F) | 0.6 |
| GC enhancer | 1.5 |
| Ultrapure H_2_O | 2.9 |
| Sample | 4 |
| Final volume | 12 |

**Table S5:** dPCR protocol for *E. coli* and *S. epidermidis*

| **Step** | | **Temperature** | **Time** |
| --- | --- | --- | --- |
| PCR Initial Heat Inactivation | | 95ºC | 2 min |
| Denaturation | 40x | 95ºC | 15 s |
| Combined annealing / extension |  | 58ºC | 1 min |

**Table S6:** Pickering Labs Artificial Saliva Composition

| **Component** | **Chemical Formula** | **Molar mass (g/mol)** | **Concentration (g/L)** | **Molality**  **(mol / kg)** |
| --- | --- | --- | --- | --- |
| Sodium chloride | NaCl | 58.44 | 0.88 | 0.015 |
| Monopotassium phosphate | KH_2_PO_4_ | 136.09 | 0.21 | 0.0016 |
| Potassium chloride | KCl | 74.55 | 1.04 | 0.014 |
| Sodium bicarbonate | NaHCO_3_ | 84.01 | 0.42 | 0.0050 |
| Calcium chloride monohydrate | CaCl_2_ * H_2_O | 129.00 | 0.13 | 0.0010 |
| Ammonium chloride | NH_4_Cl | 53.49 | 0.11 | 0.0021 |
| Dipotassium phosphate | K_2_HPO_4_ | 174.18 | 0.43 | 0.0025 |
| Potassium thiocyanate | KSCN | 97.18 | 0.19 | 0.0020 |
| Magnesium chloride heptahydrate | MgCl_2_ * 7H_2_O | 221.32 | 0.04 | 0.00018 |
| Urea | CH_4_N_2_O | 60.06 | 0.12 | 0.0020 |

**Table S7:** Observed versus model droplet efflorescence times

| **RH (%)** | **Bacterium** | **Observed Efflorescence Time (min)** | **Model Equilibrium Time (min)** |
| --- | --- | --- | --- |
| 30 | *E. coli* | 15.6 ± 3.7 | 13.5 |
|  | *S. epidermidis* | 9.5 ± 2.7 |  |
| 50 | *E. coli* | 17.1 ± 3.1 | 18.6 |
|  | *S. epidermidis* | 14.2 ± 4.5 |  |
| 70 | *E. coli* | *not observable* | 30.9 |
|  | *S. epidermidis* | *not observable* |  |

**References**

1. Luo B., Schaub A., Glas I., Klein L.K., David S.C., Bluvshtein N., Violaki K., Motos G., Pohl M.O., Hugentobler W., Nenes A., Krieger U.K., Stertz S., Peter T., Kohn T. 2023. Expiratory aerosol pH: the overlooked driver of airborne virus inactivation. Environ Sci Technol 57:486–497.
2. Schaub A., Luo B., David S.C., Glas I., Klein L.K., Costa L., Terrettaz C., Bluvshtein N., Motos G., Violaki K., Pohl M.O., Hugentobler W., Nenes A., Stertz S., Krieger U.K., Peter T., Kohn T. 2024. Salt supersaturation as an accelerator of influenza A virus inactivation in 1 μL droplets. Environ Sci Technol 58:18856–18869.
3. Krieger U.K., Siegrist F., Marcolli C., Emanuelsson E.U., Gøbel F.M., Bilde M., Marsh A., Reid J.P., Huisman A.J., Riipinen I., Hyttinen N., Myllys N., Kurtén T., Bannan T., Percival C.J., Topping D. 2018. A reference data set for validating vapor pressure measurement techniques: homologous series of polyethylene glycols. Atmos Meas Tech 11:49–63.
4. Hamer W.J., Wu Y.-C. 1972. Osmotic coefficients and mean activity coefficients of uni-univalent electrolytes in water at 25°C. J Phys Chem Ref Data 1:1047–1106.
